# Covalently modified magnetite nanoparticles with PEG: preparation and characterization as nano-adsorbent for removal of lead from wastewater

**DOI:** 10.1186/2052-336X-12-103

**Published:** 2014-08-04

**Authors:** Nejat Sadati Behbahani, Kobra Rostamizadeh, Mohammad Reza Yaftian, Abbasali Zamani, Hamideh Ahmadi

**Affiliations:** 1 Phase Equilibria Research Laboratory, Department of Chemistry, Faculty of Science, University of Zanjan, 45371-38791 Zanjan, Iran; 2 Zanjan Pharmaceutical Nanotechnology Research Center, Department of Medicinal Chemistry, School of Pharmacy, Zanjan University of Medical Sciences, Postal Code 45139-56184 Zanjan, Iran; 3 Environmental Science Research Laboratory, Department of Environmental Science, Faculty of Science, University of Zanjan, 45371-38791 Zanjan, Iran

**Keywords:** Nanotechnology, Nano-adsorbent, Magnetite, Wastewater, Response surface methodology

## Abstract

**Background:**

Lead is one of the hazardous materials which is associated with pollution and toxicity problems. This paper describes a novel approach for removal of lead from wastewater. Although naked magnetic nanoparticles have been applied for removal of different pollutants from wastewater, there was no research on employment of covalently PEG modified magnetic nanoparticles for such purpose.

**Results:**

A magnetic nano-adsorbent was prepared by chemically modification of magnetite nanoparticles (MNPs) with polyethylene glycol (PEG) for removal of lead ions. The surface of MNPs was coated covalently with 3-aminopropyltriethoxysilane (APTES) and PEG. Modified MNPs (MNPs-APTES-PEG) were characterized by FT-IR, XRD, SEM, and particle size analysis. Compared to the oleic acid coated MNPs, MNPs-APTES-PEG exhibited significant higher uptake capability for Pb(II) ions. The effective parameters on the extent of adsorption (time, temperature, Pb(II) concentration, contact time and pH) were studied and optimized by response surface methodology. Maximum uptake of MNPs-APTES-PEG for Pb(II) was determined to be 81.39 ± 2.5%. The results showed that the kinetic data was best described by Pseudo-second order model as evidenced by the relatively high value of determination coefficient (R^2^ = 0.9998). Successful removal of Pb(II) from industrial wastewater was also accomplished by MNPs-APTES-PEG.

**Conclusions:**

The results revealed high capability and excellent efficiency of developed nano-adsorbents in removal of lead contaminants from industrial wastewater.

## Background

The environment and all the life on earth are being confronted with a very serious threat as a result of high levels of pollution due to rapid industrialization. Unlike organic pollutants, the majority of which can be degraded biologically, metal ions do not easily get converted into harmless end products. Among various pollutants, heavy metal ions like Cd, Zn, Hg, Cu and Pb are commonly associated with pollution and toxicity problems. Therefore, heavy metals level in wastewater, drinking and irrigation water should be reduced to the maximum permissible concentration.

Several methods including chemical precipitation, ion-exchange process, electrolytic method, adsorption onto activated carbon, organic-based ligand precipitation, membrane, and reverse osmosis processes have been reported for elimination of these metal ions [1-4]. Most of conventional techniques are costly and have significant disadvantages such as generation of metal bearing sludge or wastes, incomplete metal removal, and disposal of secondary wastes.

Techniques based on adsorption of heavy metals have extensively attracted attention in recent years with respect to its simplicity and relatively low-cost [5-8]. Nano-adsorbents because of their extremely small size and high surface area to volume ratio, which provide better kinetics for adsorption of metal ions from aqueous solutions, have drawn particular attention recently [9-12]. However, for such an application, it is necessary to use materials that can be recycled and easily used at industrial scale.

Magnetic nanoparticles (MNPs) are a new class of nano-adsorbents which not only possess quite good performance owing to high efficient specific surface area and absence of internal diffusion resistance compared to the traditional adsorbents, furthermore can be recovered rapidly by an external magnetic field [13-23]. Kakavandi et al. [13] employed Fe_3_O_4_-activated carbon magnetic nanoparticles as an adsorbent for the removal of aniline. Nassar [21] has employed Fe_3_O_4_ nano-adsorbents for removal of Pb(II) ions from aqueous solutions by a batch-adsorption technique. The effects of contact time, initial concentration of Pb(II) ions, temperature, pH of solution and coexisting ions on the amount of Pb(II) adsorbed have been investigated.

Generally, MNPs are composed of the magnetic core and polymeric shell and their properties can be manipulated by the appropriate choice and chemical modification of polymeric shell [22]. The adsorption of Cu(II) nitrate by gum arabic modified magnetite (GA-MNP) was studied by Banerjee et al. [23]. They reported that both the naked MNP and GA-MNP could be used for the adsorption of copper ions through the complexation with the surface hydroxyl groups of MNP and the amine groups of gum arabic. A chitosan-based hydrogel, graft-copolymerized with methylenebisacrylamide and poly(acrylic acid), has been also employed in order to study the adsorption kinetics of Pb(II), Cd(II), and Cu(II) ions in aqueous solution [24]. Various parameters such as pH, initial metal concentration and extent of hydrogel mass were optimized to reach the maximum removal of metals. Mahdavian et al. [25] investigated the suitability of anchored polyacrylic acid on super paramagnetic nanoparticles for separation of heavy metal cations such as Cd(II), Pb(II), Ni(II) and Cu(II). The results showed that the capacity of cation separation is reduced only 4-6% during each recycling step which can be related to the stability of modified magnetic nanoparticles after separation and recycling process.

Considering the complexation capability of the cations by polyethylene glycol **(**PEG**)**, it seems that PEG is a promising candidate to modify magnetic nanoparticles in order to improve cations removal from aqueous solution. To the best of our knowledge, there is no report on application of PEG modified MNPs for wastewater treatment. In this contribution, a novel magnetic nano-adsorbent was developed for the adsorption of lead ions by the chemically modification of MNPs with 3-aminopropyltriethoxysilane (APTES) and PEG. The characterization of the modified-MNPs was carried out by scanning electron microscopy (SEM), and fourier-transform infrared (FT-IR) Spectroscopy. The adsorption capability of modified-MNPs was investigated using Pb(II) because of its extensive environmental impacts.

## Methods

### Materials

Analytical grade ferric chloride hexahydrate (FeCl_3_·6H_2_O), ferrous chloride tetrahydrate (FeCl_2_·4H_2_O), ammonium hydroxide (25 wt% NH_3_ in water), oleic acid (90%), ethanol, Polyethyleneglycol with molecular weight of 1000, 2000, 4000, and 6000 g/mol **(**PEG**),** 3-aminopropyltriethoxysilane (APTES), succinicanhydride, 4-(Dimethylamino)-pyridine(DMAP), triethylamine, Cu(NO_3_)_2_ 3H_2_O, Pb(NO_3_)_2_ and the chloride salts of other metals, including Ni(II), Co(II), Zn(II) were from Merck and purchased locally and used as received. 1,4-dioxane was obtained from Fluka.

### Synthesis of PEG modified magnetite nanoparticles (PEG-APTES-MNPs)

**Figure 1:**
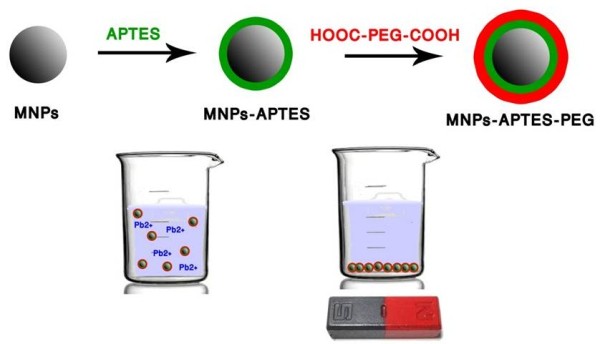
Synthesis of MNPs and their surface modification by APTES and HOOC-PEG-COOH.

The synthetic path for preparation of MNPs-APTES-PEG is schematically shown by Figure 1 and will be described in detail below:

### Synthesis of oleic acid coated magnetite nanoparticles (MNPs-OA)

MNPs-OA were prepared by the method developed by Liu et al. [26]. Briefly, 11.60 g FeCl_3_ .6H_2_O and 4.30 g FeCl_2_.4H_2_O were dissolved in 400 ml de-ionized water under nitrogen gas with vigorous stirring at 90°C. 15 ml of 25 wt% NH_4_OH was added to the solution. Then, 9 ml oleic acid was added dropwise into the suspension. After several minutes, the upper solution became colorless and the tarlike black magnetic gel precipitated and was isolated. MNPs were thoroughly washed with ethanol or acetone to remove excess oleic acid. In synthesis of physically modified MNPs with PEG (MNPs(PEG)), PEG was used instead of oleic acid, and the ratio of PEG to iron cations was set to 2:1.

### Surface coating of MNPs by APTES (APTES- MNPs)

4.22 g MNPs were dispersed in 150 ml ethanol/water (volume ratio, 1:1) solution. Then 16.16 g of APTES was added to the solution under N_2_ atmosphere at 40°C for 2 h. The prepared MNPs-APTES were collected with a magnet, and washed with ethanol and then with de-ionized water for three times. Finally, MNPs-APTES were dried under vacuum at 70°C [13].

### Synthesis of dicarboxylated PEG (HOOC-PEG-COOH)

Various M_W_ of PEG (5 g), succinic anhydride (1 g), DMAP (0.6 g) and triethylamine (0.7 ml) were dissolved in 1,4-dioxane (50 ml) and left overnight at room temperature. The filtered solution was precipitated by adding ether, and the polymeric precipitate was dried under vacuum at room temperature.

### Surface modification of MNPs-APTES by dicarboxylated PEG

To synthesize PEG functionalized MNPs, the APTES coated magnetite particles were mixed with the HOOC-PEG-COOH in the ratio of 1:2 and stirred overnight at 60°C under N_2_ protection. The resulting solid particles were dried in vacuum for further experiments.

### Characterization

The morphology of MNPs-APTES-PEG was observed by SEM (model XL30; Philips, Eindhoven, The Netherlands). X-ray diffraction (XRD) measurement was performed on a X-ray diffractometer (SIEMENS, D5000 (GERMANY)) using Cu radiation (λ = 0.1542 nm). FT-IR spectra were recorded on a Bruker, Tensor 27 (GERMANY). The particle size (z-average) of nanoparticles was determined by photon correlation spectroscopy (PCS) by a Malvern Nano/zetasizer (Malvern Instruments, UK).

### Adsorption studies

Batch adsorption tests were conducted in order to study the adsorption behavior of the MNPs-APTES-PEG toward Pb(II). In essence, a known amount of MNPs-APTES-PEG was mixed with 20 ml of Pb(II) solution over a period of time at 10–40°C on a shaker at 200 rpm, and pH of solution was adjusted by adding 0.1 M NaOH or 0.1 M HCl. Magnetic nanoparticles were separated magnetically and concentration of Pb(II) in the solution was determined using an atomic absorption spectrometer (Varian, model AA-220, Australia). The uptake percentage of Pb(II) and equilibrium adsorption capacity, Q_e_, were calculated according to the following equations, respectively:

(1) $$\mathrm{Uptake}\;\mathrm{percentage}\;=\;\frac{C_{0}-C_{e}}{C_{0}}\times 100$$

(2) $$Q_{e}=\left(C_{0}‒C_{e}\right)\frac{V}{m}$$

Where *C*_0_ is the initial Pb(II) concentration (mg*/*L), C_e_ is the Pb(II) equilibrium concentration in the aqueous solution (mg*/*L), V is the volume of the aqueous solution (L), and m is the weight of the sorbent (g).

### Optimization of Pb(II) uptake on MNPs-APTES-PEG

Pb(II) uptake on MNPs-APTES-PEG is assumed to be affected from several independent variables. In order to reach the maximum adsorption capacity, it is vital to determine the values of these variables at the optimum function. One of the well established approaches to achieve this objective is to use experimental design in conjunction with response surface methodology (RSM). In this study, uniform design (UD) and RSM were used to optimize uptake extent of Pb(II) on MNPs-APTES-PEG.

### Process variables and experimental design

Among different factors affecting the uptake efficiency, impact of main determining process variables, viz. initial Pb(II) concentration (C_0_), molecular weight of PEG (M), initial pH of solution (pH), contact time (t), and temperature (T) on uptake of Pb(II) by the MNPs-APTES-PEG in aqueous medium were investigated. Based on uniform design, 24 runs for optimization of five process variables at four levels were conducted. Range and level of independent variables are shown in Table 1.

**Table 1:** List of variables and their level

**Level**	**PEG (g mol**^**-1**^**)**	**[Pb(II)] (mgL**^**-1**^**)**	**pH**	**Time (min)**	**Temp. (**°**C)**
**1**	1000	10	3	10	10
**2**	2000	20	4	20	15
**3**	4000	30	5	30	25
**4**	6000	40	5.5	40	35

### Response surface modeling

An empirical quadratic equation model for five parameters was used to model the adsorption process (Eq. 3):

(3) $$\begin{array}{ll} Y= & \beta_{0}+\;\beta_{1}X_{M}+\beta_{2}X_{C}+\beta_{3}X_{\mathrm{pH}}+\\ & \beta_{4}X_{t}+\beta_{5}X_{T}+\beta_{6}X_{M}^{2}+\beta_{7}X_{C}^{2}+\beta_{8}X_{\mathrm{pH}}^{2}+\beta_{9}X_{t}^{2}\\ & +\beta_{10}X_{T}^{2}+\beta_{12}X_{M}X_{C}+\beta_{13}X_{M}X_{\mathrm{pH}}\\ & +\beta_{14}X_{M}X_{t}+\beta_{15}X_{M}X_{T+}\beta_{16}X_{C}X_{\mathrm{pH}}\\ & +\beta_{17}X_{C}X_{t}+\beta_{18}X_{C}X_{T}+\beta_{19}X_{\mathrm{pH}}X_{t}\\ & +\beta_{20}X_{\mathrm{pH}}X_{T}+\beta_{21}X_{t}X_{T} \end{array}$$

The model coefficients (β_i_) were calculated and used for prediction of the response values for different combinations of variables. The quadratic model equation was solved with the help of the Design Expert V.6.0.7 software (Stat-Ease Inc., USA).

### Statistical analysis

The adequacy of developed model, the significance of independent variables and their interactions were analyzed by means of the analysis of variance (ANOVA). Statistical analysis was performed using Design Expert V.6.0.7. The variables were considered as the significant factor when p-value < 0.05. The optimal values of the operation parameters were estimated by the three-dimensional response surface analysis of the independent variables. MATLAB (version 6.5) was used for graphical analysis of the data.

### Adsorption kinetics

Adsorption is time-dependent process and it is of critical importance to know the rate of adsorption in order to design potential adsorbent for removal of lead. The experimental data were applied to the pseudo-first-order (Eq. 4), pseudo-second-order (Eq. 5), simple Elovich (Eq. 6) and power function (Eq. 7) kinetic models.

(4) $$\log\left({q‒\;q}_{e}\right)={\mathrm{logq}}_{e}‒\frac{K_{1\;}}{2.303}t$$

(5) $$\frac{t}{q}=\frac{1}{K_{2\;}q_{e}^{2}}+\;\frac{1}{q_{e}}t$$

(6) q=a+2.303blogt

(7) logq=loga+blogt

where q_e_ and q are the amount of Pb(II) adsorbed on the adsorbent in mg/g at equilibrium and at time t, respectively, and K_1_, K_2_, a, and b are the constant parameters of the corresponding models and were calculated by least-squares regression analysis to evaluate the applicability of the kinetic models to fit the experimental data.

### Desorption experiments

In order to estimate the recovery of Pb(II) from MNPs-APTES-PEG, desorption experiments with different stripping solutions (H_2_SO_4_, HCl and HNO_3_ solutions) at various concentrations were performed. 20 ml of Pb(II) solution with initial concentration of 35 mg/l and 0.05 g of MNPs-APTES-PEG was shaken at 150 rpm and 10°C. After 35 min the adsorbents were separated magnetically and were added to 20 ml of the effluent. Samples were analyzed to evaluate metal recovery. The metal recovery was calculated by the following equation:

(8) $$R\left(\%\right)=\frac{m_{\mathrm{des}}}{m_{\mathrm{ads}}}\times 100$$

Where m_des_ and m_ads_ are the amount of Pb(II) released into the aqueous solution and the amount of Pb(II) adsorbed onto the MNPs-APTES-PEG nano-adsorbents (mg), respectively.

### Application of MNPs-APTES-PEG in real samples

To demonstrate the potential removal efficiency of MNPs-APTES-PEG in adsorption of Pb(II) from solutions containing other metal ions, the modified nano-adsorbent was employed for removal of Pb(II) from industrial wastewater. Industrial wastewater samples were collected from the Zanjan Zinc Industries Company, Zanjan, Iran which was composed of Ni(II), Cd (II), Co(II),Pb(II), Cu(II), Zn(II) with the concentration value of 42.76, 258.75, 7.893, 6.84, 0.655, 18370 mg L^-1^, respectively. All experiments carried out under optimal condition for maximum uptake of Pb(II). Three replicates were carried out on a sample.

## Results and discussion

### Characterization of the PEG-APTES-MNPs

**Figure 2:**
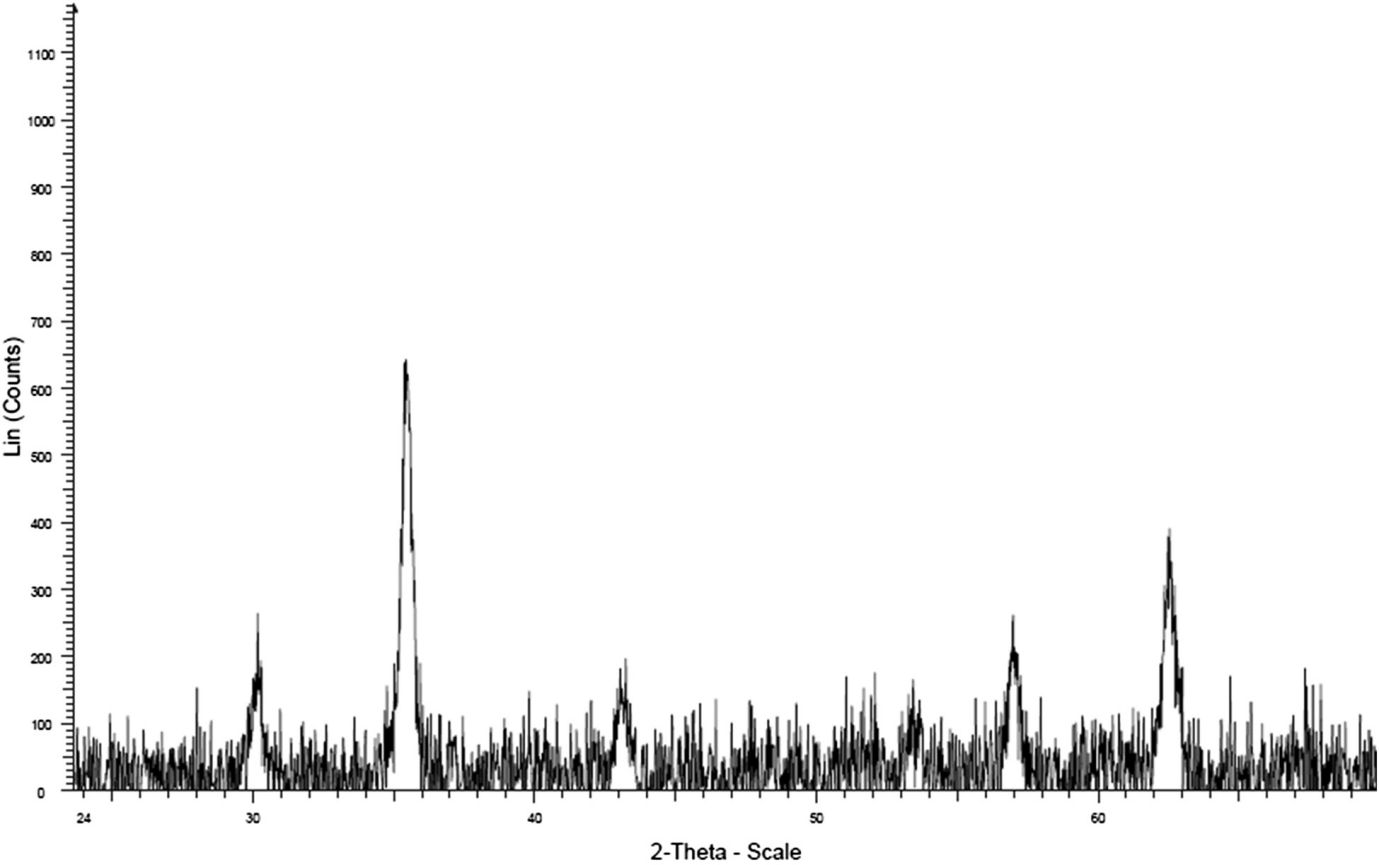
XRD pattern of MNPs sample.

**Figure 3:**
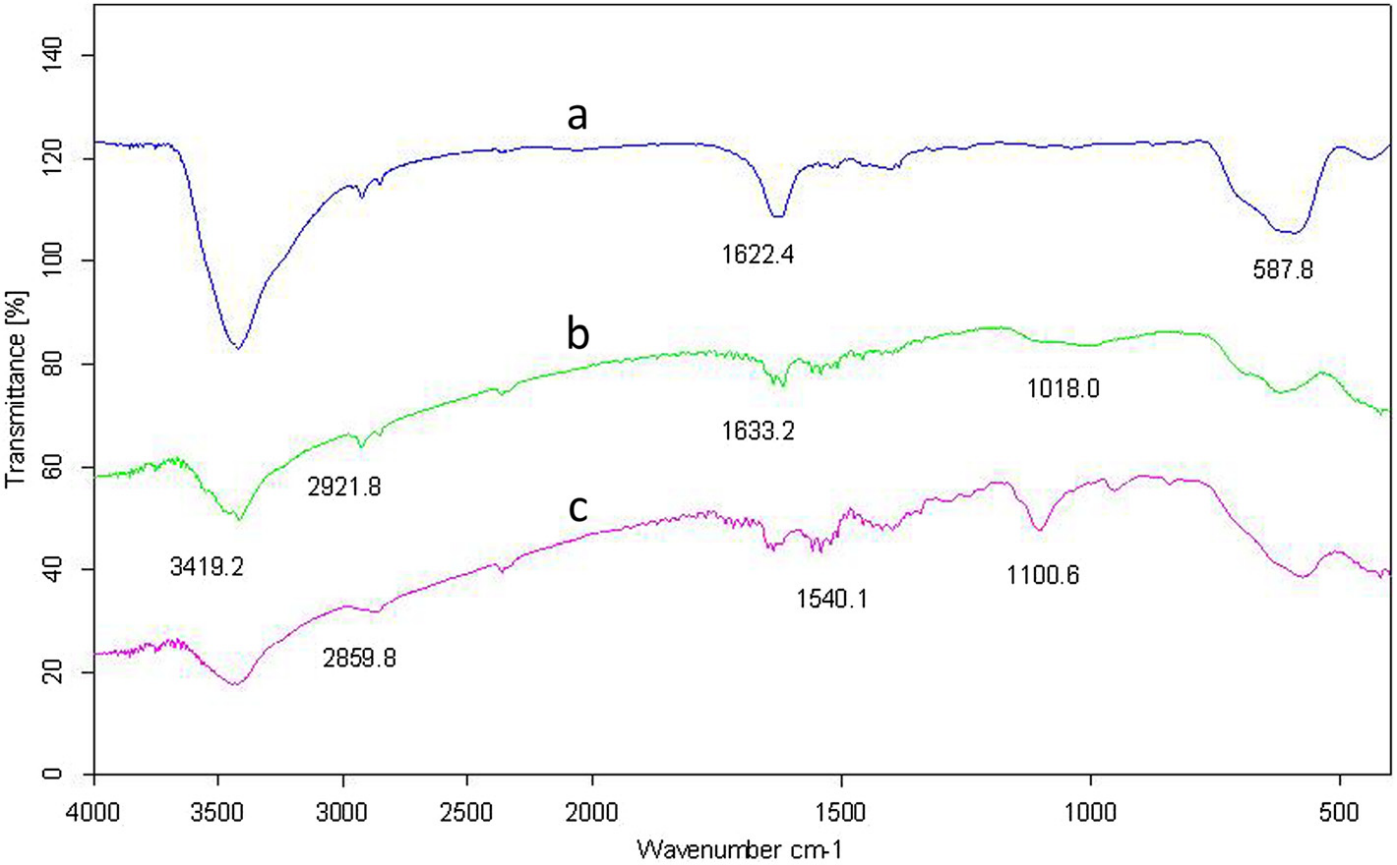
FTIR spectra of MNPs (a), MNPs-APTES (b), and MNPs-APTES-PEG (c).

Synthesis of Fe_3_O_4_ was confirmed by XRD spectrum (Figure 2). The result showed that diffraction patterns and relative intensities of all diffraction peaks conform well to those of magnetite. The chemical structure of the MNPs-OA, MNPs-APTES and MNPs-APTES-PEG were characterized by FT-IR (Figure 3). The adsorption peaks at around 587.8 and 1622.4 cm^-1^ in MNPs-OA spectrum were the characteristic absorption of Fe–O and C=O bonds which confirmed the presence of magnetite nanoparticles covered by oleic acid (Figure 3a). The introduction of APTES to the surface of MNPs was confirmed by the bands at 1018.0 cm^-1^ assigned to the Si–O group (Figure 3b). The two bands at 3419.2 and 1633.2 cm^-1^ can be referred to the N–H stretching vibration and NH_2_ bending mode of free NH_2_ group, respectively. The presence of the anchored propyl group was confirmed by C–H stretching vibrations that appeared at 2921.8 cm^-1^.

**Figure 4:**
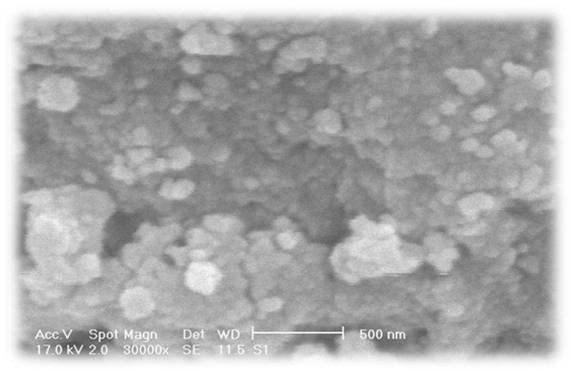
SEM image of MNPs-APTES-PEG.

Successful synthesis of dicarboxylated PEG was demonstrated by the appearance of the characteristic absorption peaks of 1731.9 and 3461.8 cm^-1^ corresponding to C = O and -OH bonds, respectively, which confirmed formation of carboxylic group in the dicarboxylated PEG [18]. The MNPs-APTES were treated with HOOC–PEG-COOH to give MNPs-APTES-PEG. The appearance of band at about 1540.1 cm^-1^, indicative of the –C(=O)–N–H vibration, confirmed the amide bond formed between amine groups on the surface of the APTES-MNPs and carboxyl groups in PEG (Figure 3c). Moreover, the characteristic bands of PEG at 2859.8 cm^-1^ (C–H symmetric stretching) and 1100.6 cm^-1^ (C–O stretching) further support the attachment of PEG onto the MNPs-APTES surface. Modification of MNPs by amorphous PEG was also confirmed by X-ray technique, as evidenced by disappearance of characteristic bands at X-ray spectrum which can be indicative of the presence of an amorphous state at the surface of MNPs (data not shown).Using scanning electron microscopy, it is possible to obtain information about the surface properties of the nano-adsorbents (Figure 4). In accordance with the scanning electron microscopy it can be postulated that the nanoparticles were in uniform spherical shape. The hydrodynamic diameter of particles which were determined by PCS was found to be 124.1 nm (z-average) with polydispersity index of 0.685.

### Adsorption capability

As a preliminary study, the capability of the MNPs-OA, and physically modified MNPs-PEG was compared with MNPs-APTES-PEG magnetic nano-adsorbents in terms of removal of Pb(II) from aqueous solution (Table 2). It is clear that MNPs-APTES-PEG offer a significantly higher capability for adsorbtion of Pb(II) in comparison with the MNPs-OA and physically modified MNPs(PEG). This remarkable performance would be ascribed from covalently attached PEG which provides more adsorption sites for cations. So it can be concluded that MNPs-APTES-PEG can be considered as a promising candidate for removal of Pb(II) from aqueous solutions.

**Table 2:** Recovery of Pb (II) from aqueous solutions by MNPs-OA, physically modified MNPs (PEG), and MNPs-APTES-PEG nano-adsorbent

	**MNPs**	**physically modified PEG-MNPs**	**MNPs-APTES-PEG**
**Pb(II) Uptake%**	5.5	7.2	37

Experimental conditions: contact time 35 min, Pb(II) concentration = 35 mg L^-1^, shaking rate: 1000 rpm, volume of feed solution 20 ml, pH =5.5, amount of adsorbent 0.05 g, Temperature 10°C.

### Optimization of adsorption uptake

In order to enhance the adsorption capacity of MNPs-APTES-PEG, the individual and interactive effects of the five important variables including initial Pb(II) concentration (C_o_), molecular weight of PEG (M), initial pH of solution (pH), contact time (t), and temperature (T) on the uptake of Pb(II) were investigated.

### Experimental design and regression model

Uniform design was employed as experimental design approach to study the individual and interactive effect of selected variables on the uptake of Pb(II) by MNPs-APTES-PEG. The order of experiments was arranged randomly in order to minimize the effects of uncontrolled factors (Table 3).

**Table 3:** Experimental designs matrix

**Run**	**PEG (g mol**^**-1**^**)**	**Pb(II) (mg L**^**-1**^**)**	**pH**	**Time (min)**	**Temp. (°C)**
**1**	6000	20	3	20	10
**2**	6000	30	3	40	25
**3**	2000	30	4	40	15
**4**	1000	40	3	20	15
**5**	6000	20	5.5	30	35
**6**	2000	20	5	10	25
**7**	4000	40	5.5	40	25
**8**	2000	10	5	40	35
**9**	1000	30	5	30	10
**10**	4000	40	5	20	25
**11**	6000	30	4	10	35
**12**	6000	40	5	30	10
**13**	1000	20	3	10	25
**14**	4000	20	4	40	10
**15**	6000	10	5	10	15
**16**	2000	40	4	10	10
**17**	1000	30	5.5	20	35
**18**	4000	10	4	20	35
**19**	1000	10	4	30	25
**20**	2000	40	3	30	35
**21**	1000	20	5.5	40	15
**22**	2000	10	5.5	20	10
**23**	4000	30	5.5	10	15
**24**	4000	10	3	30	15

A quadratic model was selected to correlate relationship between the response (Y: uptake percentage of Pb(II)) and the process variables (X: T, pH, C, t, M). The best empirical model in terms of independent variables was obtained as follow (Eq. 9):

(9) $$\begin{array}{ll} Y= & -339.78+36.69X_{M}+4.54X_{C}+73.57X_{\mathrm{pH}}\\ & -0.42X_{t}+8.81X_{T}-3.46X_{M}^{2}+0.044X_{C}^{2}\\ & -5.09X_{\mathrm{pH}}^{2}-0.051X_{t}^{2}-0.14X_{T}^{2}\\ & -0.11X_{M}X_{C}-1.04X_{M}X_{\mathrm{pH}}+0.071X_{M}X_{t}\\ & -0.18X_{M}X_{T}-1.41X_{C}X_{\mathrm{pH}}\\ & +0.00734X_{C}X_{t}-0.057X_{C}X_{T}\\ & +0.79X_{\mathrm{pH}}X_{t}+0.12X_{\mathrm{pH}}X_{T}-0.033X_{t}X_{T} \end{array}$$

The model was used to evaluate the influence of the process variables on the uptake of Pb(II). ANOVA results of the model indicated that the model was highly significant, as the *F* value for the model was 15.40 which was larger compared with the critical F value (F_0.05, 14, 11_ = 2.565) (Table 4). It suggests that the computed Fisher's variance ratio at this level was large enough to justify a very high degree of adequacy of the quadratic model.

**Table 4:** Analysis of variance (ANOVA) of the response surface quadratic model

**Source**	**Sum of squares**	**Degrees of freedom**	**Mean square**	**F Value**	**P value**	**Remarks**
**Model**	10608.27	20	530.41	15.40	0.0222	Significant
**Main effects**						
**M**	659.58	1	659.58	19.15	0.0221	Significant
**C**_**0**_	204.74	1	204.74	5.94	0.0927	
**pH**	192.79	1	192.79	5.60	0.0989	
**t**	5.19	1	5.19	0.15	0.7238	
**T**	387.49	1	387.49	11.25	0.0439	Significant
**2-way interactions**						
**M×M**	864.24	1	864.24	25.09	0.0153	Significant
**C**_**0**_**× C**_**0**_	126.31	1	126.31	3.67	0.1514	
**pH× pH**	72.90	1	72.90	2.12	0.2417	
**t×t**	196.94	1	196.94	5.72	0.0966	
**T×T**	533.55	1	533.55	15.49	0.0292	Significant
**M× C**_**0**_	28.54	1	28.54	0.83	0.4298	
**M× pH**	52.67	1	52.67	1.53	0.3042	
**M×t**	19.58	1	19.58	0.57	0.5057	
**M×T**	126.64	1	126.64	3.68	0.1510	
**C**_**0 **_**×pH**	1664.09	1	1664.09	48.32	0.0061	Significant
**C**_**0 **_**×t**	4.94	1	4.94	0.14	0.7300	
**C**_**0 **_**×T**	352.99	1	352.99	10.25	0.0493	Significant
**pH×t**	421.40	1	421.40	12.24	0.0395	Significant
**pH×T**	7.15	1	7.15	0.21	0.6796	
**t×T**	84.80	1	84.80	2.46	0.2146	
**Residual**	**103.33**	**3**	**34.44**			
**Cor Total**	**10711.59**	**23**				

**Figure 5:**
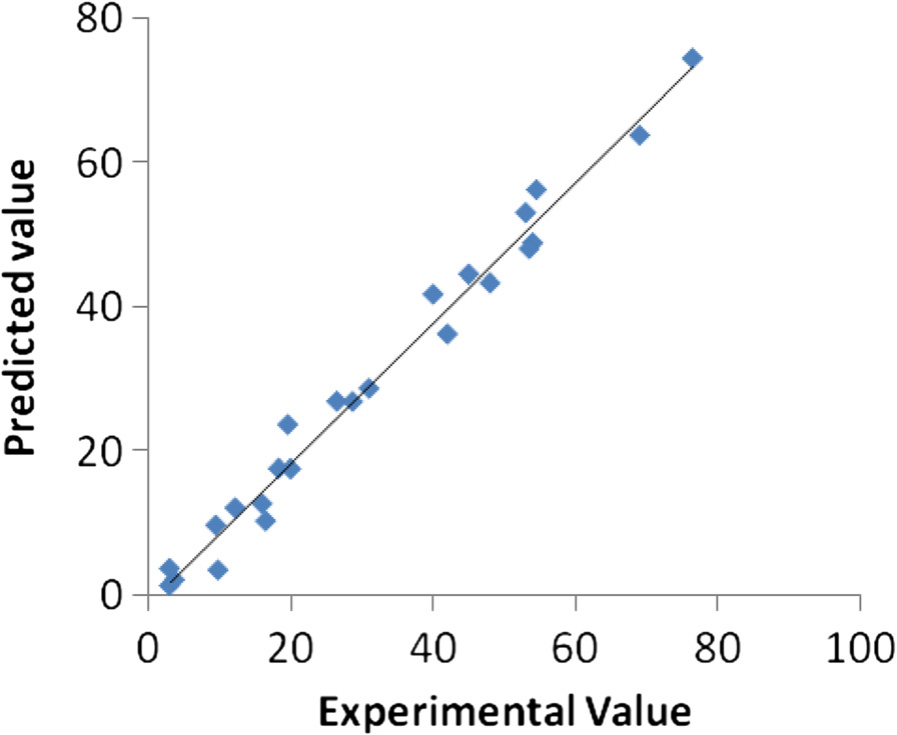
Predicted versus experimental Pb(II) uptake values.

The goodness of fit of the model was checked by the correlation coefficient (R^2^) between the experimental and model predicted values of the response variable (Figure 5). It is obvious that a high value of R^2^ (0.98) confirms the fitness of the model where 98% of the total variation in the response can be explained by the developed model, and only 2% are being explained by the residues.

A closely high value of the adjusted correlation coefficient (R^2^ adj. =0.93) also suggests that the model possess a high significance. The R^2^ adj. corrects the R^2^ value for the sample size and the number of terms in the model. If there are many terms in a model with a small sample size, R^2^ adj. may be noticeably smaller than R^2^. In our case, the values of R^2^ adj. and R^2^ were found to be close. It shows that total variation of about 93% for Pb(II) uptake capability was attributed to the independent variables while only about 7% of the total variation cannot be explained by the model. Therefore, this empirical second order quadratic equation can be applied to predict Pb(II) uptake within the experimental range.

### Impact of the model components and their interactions on Pb(II) uptake

The significance of the quadratic model coefficients was evaluated by p-values listed in Table 4. The p-value is used as a tool to check the significance of the coefficients. The smaller p-value, the more significant is the corresponding parameter in the regression model. From Table 4, it is evident that the independent variables including M, second order effect of M (M×M), T, second order effect of temperature (T×T), interactions between Pb(II) concentration (C_0_) and pH (C_0_×pH), interactions between Pb(II) concentration (C_0_) and temperature (C_0_×T), and interactions between pH and time (pH×t) were significant parameters because of *p <* 0.05. The other variables and interactions were insignificant.

### Three-dimensional response surface plots

**Figure 6:**
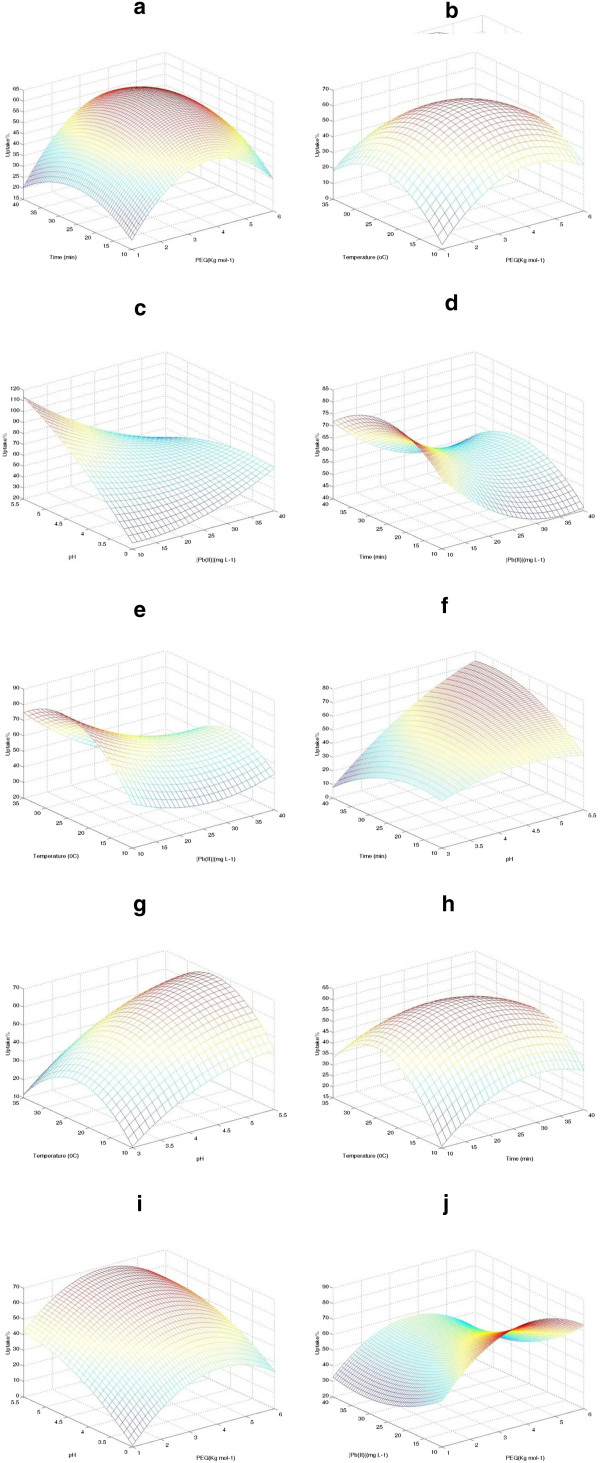
**Three dimensional figures.** The 3-D plot of Pb(II) uptake predicted by the RSM model for (a) time and PEG (M), (b) temperature and PEG (M), (c) pH and Pb(II) concentration, (d) time and Pb(II) concentration, (e) temperature and Pb(II) concentration, (f) time and pH, (g) temperature and pH, (h) temperature and time, (i) pH and PEG (M), and (q) Pb(II) concentration and PEG M. In each figure, the remaining factors were kept constant at middle level values.

Three-dimensional surfaces plots are graphical representation of regression equation and are the most useful approach in revealing the optimum condition. Indeed, each plot represents an infinite number of combinations of two tested variables with the other variables maintained at their respective middle level. The influence of five different process variables on the response factor (Pb(II) uptake) was visualized in the 3D response surface plots (Figure 6 a–q). Since, the quadratic model in this study had five independent variables, three variables were held constant at their middle level for each plot and subsequently, a total of ten 3D plots were drawn for the responses.

Optimization of the independent variables to maximize the Pb(II) uptake efficiency of the developed MNPs-APTES-PEG based on RSM leads us to conclude that the optimum condition for the Pb(II) uptake can be considered as pH value of 5, initial Pb(II) concentration of 11 mg L^-1^, temperature of 25°C, PEG molecular weight of 2000 g/mol^-1^ and contact time of 30 min (Figure 6 a–q).

### Verification of optimization condition for Pb (II) uptake

To confirm the model adequacy for predicting maximum uptake of Pb(II), the model was validated by carrying out some experiments under the optimum condition. The corresponding experimental value of the Pb(II) uptake under the optimum condition of the variables was determined as 81.39 ± 2.5%, which was well consistent with the theoretically optimized value (88.83%). This result confirms the validity of the optimal point. The reason behind such a high Pb(II) uptake probably lies in the high number of etheric groups existing in PEG backbone which provide more active sites to interact with Pb(II).

### Adsorption kinetics

**Figure 7:**
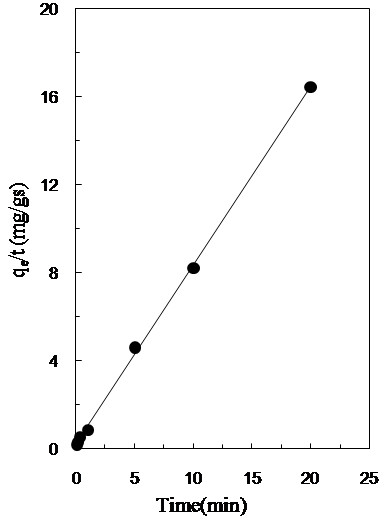
**Pseudo-second-order kinetic model for Pb(II) uptake by MNPs-APTES-PEG.** Experimental conditions: contact time: 35 min, Pb(II) concentration = 35 mg/L, shaking rate: 1000 rpm, volume of feed solution 20 mL, pH =5.5, amount of MNPs-APTES-PEG 0.05 g, Temperature 10°C.

The adsorption kinetic characteristics of the nanoadsorbant was studied using four different adsorption kinetic models frequently used in the characterization of this kind of adsorbants including pseudo first-order, pseudo-second-order, simple Elovich and power function. To distinguish the model that describes the data properly and fit the data correctly, a relatively high value of the coefficient of determination (R^2^) as well as low p-value (considering the mean of predictive error) was taken as criterion. The estimated model and related kinetic parameters are listed in Table 5. It can be seen from Table 5 that kinetic of Pb(II) uptake onto MNPs-APTES-PEG nanoadsorbent can be best described by the ‘pseudo-second order kinetic model, as shown in Figure 7.

**Table 5:** Parameters of various kinetics models fitted to experimental data

**No.**	**Kinetic model**	**R**^**2**^	**p-value**
1	Pseudo-first order	0.9916	3.6×10^-1^
2	Pseudo-second order	0.9993	5.2×10^-9^
3	Simple Elovich	0.9893	4.3×10^-5^
4	Power function	0.9808	1.3×10^-4^

### Recovery of Pb (II)

Recovery of Pb(II) has two advantages, namely valuable Pb(II) ions can be recovered and nano-adsorbents can be re-used for another cycle. The separation of Pb(II) from MNPs-APTES-PEG was accomplished using different stripping acidic solutions including H_2_SO_4_, HCl and HNO_3_ (Table 6). High recovery percentage of Pb(II) by all acidic stripping solutions can be indicative of highly dependent of desorption process on pH. On the other hand, from the results in Table 6, it is concluded that the adsorption of Pb(II) onto MNPs-APTES-PEG nano-adsorbents is reversible, and the bonding between the active sites and the adsorbed Pb(II) is not strong. The results also suggest that the Pb(II)-loaded nano-adsorbents can be easily desorbed using a very low concentration of acidic solution.

**Table 6:** Recovery percentage of Pb(II) using different stripping solutions

**Stripping solution**	**HCl**	**H**_**2**_**SO**_**4**_	**HNO**_**3**_
0.01 (M)	90.4	98.1	97.1
0.05 (M)	93.0	98.0	91.0

Experimental conditions: contact time 35 min, shaking rate: 1000 rpm, volume of feed solution 20 ml, amount of MNPs-APTES-PEG 0.05 g, temperature 23°C.

### Removal of Pb (II) from industrial wastewater

The preliminary results showed that MNPs-APTES-PEG could be a potential adsorbent to remove Pb(II) from water samples. Further applications of MNPs-APTES-PEG to remove Pb(II) in real wastewaters was also investigated. The amount of Pb(II) ion uptake percentage in industrial wastewater was found to be 62.27%. These data proved that the method is possible to achieve excellent efficiency in removal of Pb(II) from industrial wastewater by MNPs-APTES-PEG without any significant matrix effects caused by the interference of co-existing other cations.

## Conclusion

A novel magnetic nano-adsorbent has been developed by covalently modified Fe_3_O_4_ nanoparticles with PEG (MNPs-APTES-PEG). The results of FT-IR, and XRD of the MNPs before and after coating clearly indicated that coating procedure was successfully performed. Compared to the MNPs-OA and physically modified MNPs(PEG), MNPs-APTES-PEG exhibited significant high adsorption capability for Pb(II) ions. Response surface methodology was employed to search for optimal condition to reach the maximum uptake percentage. Maximum uptake of PEG–APTES-MNPs for Pb(II) under optimum condition was 81.39 ± 2.5%. The results showed that the kinetic data was best described by Pseudo-second order model as evidenced by the relatively high value of determination coefficient (R^2^ = 0.9998). The practical utility of the nano-adsorbent was demonstrated by adsorption of lead ions from industrial wastewater. These data proved that these materials hold considerable promise for the construction of new nano adsorbents with excellent efficiency in removal of Pb(II) from industrial wastewater.

## Competing interests

The authors declare that they have no competing interests.

## Authors’ contribution

NSB carried out the majority of experiments of the work and also wrote the whole article. KR, and MY gave the idea of the research and the research was done under their supervision. KR also read and revised the manuscript, HA did some experiments. AZ helped with all the experiments. All authors read and approved the final manuscript.
